# Neutralizing antibody against SARS-CoV-2 spike in COVID-19 patients, health care workers, and convalescent plasma donors

**DOI:** 10.1172/jci.insight.143213

**Published:** 2020-11-19

**Authors:** Cong Zeng, John P. Evans, Rebecca Pearson, Panke Qu, Yi-Min Zheng, Richard T. Robinson, Luanne Hall-Stoodley, Jacob Yount, Sonal Pannu, Rama K. Mallampalli, Linda Saif, Eugene Oltz, Gerard Lozanski, Shan-Lu Liu

**Affiliations:** 1 Center for Retrovirus Research,; 2 Department of Veterinary Biosciences,; 3 Molecular, Cellular and Developmental Biology Program,; 4 Department of Pathology,; 5 Department of Microbial Infection and Immunity, and; 6 Department of Medicine, The Ohio State University (OSU), Columbus, Ohio, USA.; 7 Food Animal Health Research Program, Ohio Agricultural Research and Development Center, Wooster, Ohio, USA.; 8 Viruses and Emerging Pathogens Program, Infectious Diseases Institute, OSU, Columbus, Ohio, USA.

**Keywords:** COVID-19, Cellular immune response

## Abstract

Rapid and specific antibody testing is crucial for improved understanding, control, and treatment of COVID-19 pathogenesis. Herein, we describe and apply a rapid, sensitive, and accurate virus neutralization assay for SARS-CoV-2 antibodies. The assay is based on an HIV-1 lentiviral vector that contains a secreted intron *Gaussia* luciferase (Gluc) or secreted nano-luciferase reporter cassette, pseudotyped with the SARS-CoV-2 spike (S) glycoprotein, and is validated with a plaque-reduction assay using an authentic, infectious SARS-CoV-2 strain. The assay was used to evaluate SARS-CoV-2 antibodies in serum from individuals with a broad range of COVID-19 symptoms; patients included those in the intensive care unit (ICU), health care workers (HCWs), and convalescent plasma donors. The highest neutralizing antibody titers were observed among ICU patients, followed by general hospitalized patients, HCWs, and convalescent plasma donors. Our study highlights a wide phenotypic variation in human antibody responses against SARS-CoV-2 and demonstrates the efficacy of a potentially novel lentivirus pseudotype assay for high-throughput serological surveys of neutralizing antibody titers in large cohorts.

## Abstract 2

A rapid and sensitive high-throughput neutralization assay for evaluating the neutralizing antibody levels in COVID-19-exposed individuals.

## Graphical abstract

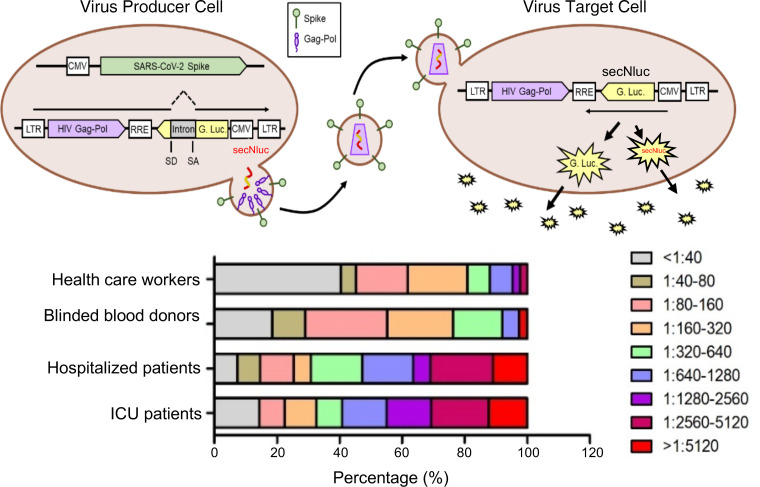

## Introduction

The COVID-19 pandemic, caused by the SARS-CoV-2 virus, which currently engulfs the globe, has led to over 10 million cases and 500,000 deaths world-wide (1, 2). Without an effective vaccine, significant public health measures are required to minimize the spread of the virus — including masks and social distancing, or measures to prevent disease transmission by providing physical distance between potential hosts — complemented with wide-spread testing and contact tracing. Reliable and easily deployed testing is necessary to fully understand the spread of the virus. Among these, antibody assays make up a critical arm of our testing capacity that will allow for large-scale serum surveillance and closer monitoring of immunity in individual patients (3–6). Even after the development of a vaccine, large-scale antibody testing is needed to track vaccine and herd immunity as SARS-CoV-2 infections subside (7, 8). This is particularly important since antibody responses to SARS-CoV-2 infection may wane over time, as is the case for other human coronaviruses (9–11). Additionally, in the short-term, convalescent plasma from recovered SARS-CoV-2 patients has been used to treat severe COVID-19 cases, and testing convalescent plasma for high SARS-CoV-2 neutralizing antibody titers can improve this treatment strategy (12–15). Thus, accurate determinations of neutralizing antibody titers are of particular importance for both surveillance and testing of patient serum.

Recent evidence has suggested that several available antibody tests exhibit poor correlation with neutralizing titers (4). Following the large SARS-CoV-2 outbreak in New York (New York, USA), several studies investigated the seroconversion of previously infected individuals (4–6). The vast majority of COVID-19 patients were shown to seroconvert by ELISAs (5). However, other analyses have demonstrated that recovered SARS-CoV-2 patients often have only weak titers of neutralizing antibodies against the virus (4, 6, 16, 17), further emphasizing the need for reliable antibody neutralization tests.

Several pseudotype virus neutralizing antibody tests have been developed for SARS-CoV-2 (18–22). To avoid the need for live virus and a BSL3 facility, these assays typically use an HIV-based or vesicular stomatitis virus–based (VSV-based) vector pseudotyped with the SARS-CoV-2 spike (S) protein (4, 20, 23). This approach allows for the accurate detection of anti-S neutralizing antibodies in a more commonly available BLS2 facility. However, existing neutralization assays use a firefly luciferase, Renilla luciferase, or GFP reporter cassette that typically require a cell-lysis step, increasing the experimental timeline and reducing the scale-up capacities. Here, we developed a SARS-CoV-2 antibody neutralization assay that uses an HIV-1 vector bearing a secreted intron *Gaussia* luciferase (Gluc or nano-luciferase [Nluc]) reporter cassette and is pseudotyped with SARS-CoV-2 S protein. This approach permits sensitive, rapid, and accurate testing of neutralizing antibody titers without a cell-lysis step, and it was validated with an authentic SARS-CoV-2 USA-WA-1 strain in plaque-reduction virus neutralization (PRVN) assays. Using our S pseudotype virus assay, we examined neutralizing antibody titers for 221 blinded serum samples, which include 104 hospitalized COVID-19 patients (49 intensive care unit [ICU] patients and 55 inpatients), 42 OSU health care workers (HCWs), 38 convalescent plasma donors (all were RT-PCR confirmed), and 37 negative control samples collected before September 2019. Our neutralization results are highly concordant with that of SARS-CoV-2 nucleocapsid–based (SARS-CoV-2 N–based) IgG antibody ELISA measurements. Prior reports are conflicting on the presence of cross-reactive antibodies to SARS-CoV and SARS-CoV-2 (24–27). In the patient samples tested, we found no antibody cross-neutralization of SARS-CoV in COVID-19 patients. Additionally, our results bolster prior reports that more severe COVID-19 cases tend to have higher neutralizing antibody titers (25) but also indicate that HCWs, as well as convalescent plasma donors, have a varied neutralizing antibody response, which will require more attention from the scientific and public health communities.

## Results

### Generation of secreted intron Gluc-based lentiviral pseudotypes bearing SARS-CoV-2 S.

**Figure 1:**
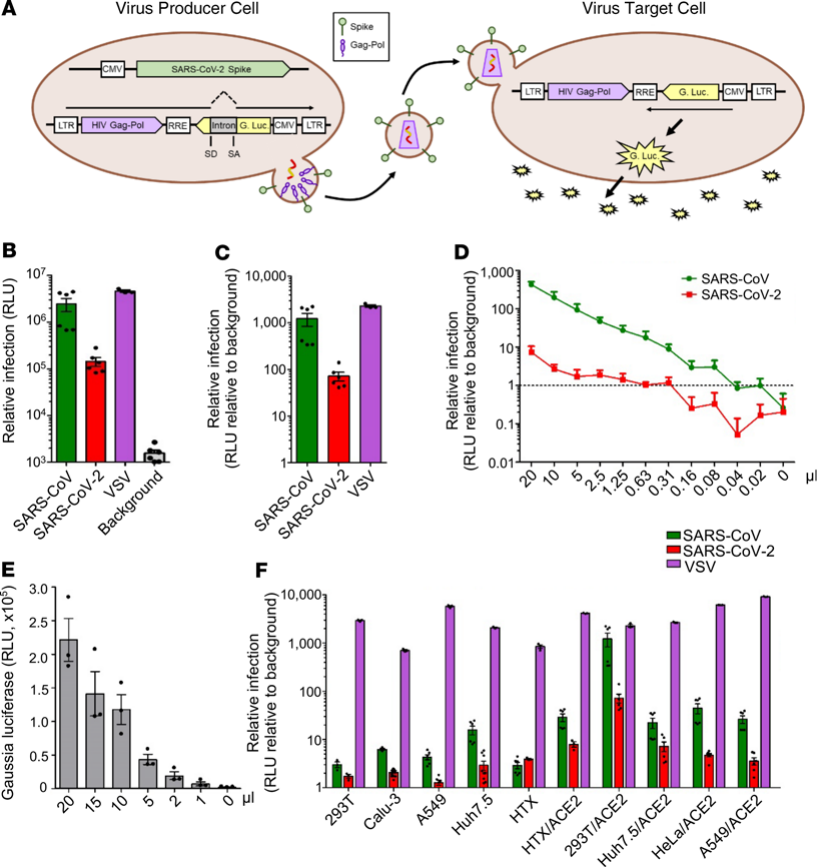
inGluc-based HIV-1 lentiviral S pseudotypes bearing SARS-CoV-2 spikes. 293T cells seeded on 6-well plates were cotransfected with 0.8 μg HIV-1–NL4.3–inGluc vector plus 0.4 μg SARS-CoV-2 spike-coding plasmid. Forty-eight hours after transfection, viral supernatant was harvested and used to infect target cells. Unless otherwise indicated, 293T/ACE2 cells were used for infection. (**A**) Schematic representation of the pseudoviral production and infection. Note that Gluc activity can only be detected in virus-infected target cells — and not in the virus-producing cells — because of the presence of an intron inserted in the sense of the vector that splits the Gluc gene into 2 parts. (**B** and **C**) Titers of HIV-1 inGluc pseudotypes bearing the spikes of SARS-CoV (*n* = 6), SARS-CoV-2 (*n* = 6), or VSV-G (*n* = 3); absolute luciferase readouts at 48 hours after infection, and relative infectivity compared with the background, were plotted, respectively. (**D**) Indicated doses of viral supernatant were used to infect 293T/ACE2 cells seeded in 24-well plates, and 20 μL of supernatant of virus-infected cells were used to measure the Gluc activity as shown. The dashed line indicates the background of luciferase activity; *n* = 3. (**E**) Indicated amounts of culture media harvested from virus-infected cells were used to measure Gluc activity; *n* = 3. (**F**) Relative infectivity of HIV-inGluc pseudotypes bearing S proteins of SARS-CoV, SARS-CoV-2, or VSV-G in indicated target cells, with parental or those overexpressing ACE2; *n* = 6. Data were analyzed as mean ± SD.

The S protein of coronavirus can pseudotype a variety of viral vector systems, including lentiviral vectors (18–20). Differing from most lentiviral pseudotypes, which bear GFP, alkaline phosphatase, or firefly/renilla/Nluc luciferases as a reporter, we chose Gluc as the transducing gene, given its natural secretion into mammalian cell culture media and high sensitivity, thus facilitating detection (28). To prevent Gluc activity in producer cells, which may cause a high background signal in the media of target cells, we took advantage of a published intron Gluc (inGluc) system (29, 30). In this lentiviral vector, the antisense Gluc reporter gene is interrupted by an intron oriented in the sense direction of the HIV-1 NL4.3 genome. As such, expression of Gluc can be detected only in vector-transduced target cells after infection (Figure 1A).

To test the HIV-1–NL4.3–inGluc vector for SARS-CoV-2, we transfected 293T cells seeded on a 6-well plate, which also expresses HIV-1 gag-pol but not Env, together with a plasmid encoding the S protein of SARS-CoV-2 that is tagged with C9 (TETSQVAPA) at the C-terminus to facilitate detection (31). In parallel, the S of SARS-CoV, which also has a C9 tag, or VSV-glycoprotein (VSV-G), were cotransfected. Supernatants from transfected cells were harvested and used to infect target 293T cells overexpressing the SARS-CoV-2 receptor angiotensin-converting enzyme 2 (ACE2) (292T/ACE2) (32, 33) in 96-well plates, and Gluc activity was measured at 24, 48, and 72 hours after infection (Figure 1B). We found that SARS-CoV-2 S protein can pseudotype the inGluc-based lentiviral vector, although the infectivity of SARS-CoV-2 S pseudotypes was 5- to 10-fold lower than that of SARS-CoV; VSV pseudotypes exhibited the highest titer, as would be expected (Figure 1B). The relative infectivity of SARS-CoV, SARS-CoV-2, and VSV pseudotypes in 293T/ACE2 cells, as compared with the background control, was plotted in Figure 1C, showing that lentiviral SARS-CoV-2 S pseudotypes consistently produced a luciferase signal 50- to 100-fold above background at 2–3 days after infection.

To gain insight into the efficiency of SARS-CoV-2 S pseudotyping and the sensitivity of this lentiviral system, we applied different amounts of viral stocks in 2-fold serial dilutions (e.g., from 20, 10, 5, and 2.5, to 0.02 μL), to target cells (Figure 1D), along with serially reduced volumes of culture media of infected cells (e.g., 20, 15, 10, 5, 2, 1 μL), to measure Gluc activity (Figure 1E). Activity can be detected even with less than 1 μL of culture media for Gluc measurements, and by calculating the minimal amount of virus stock and culture media that gave a Gluc signal above background, we were able to estimate that the infectious units (IU) of SARS-CoV-2 and SARS-CoV lentiviral Gluc pseudotypes were about 8.0 × 10^4^ mL and about 3.2 × 10^5^ per mL, respectively.

To identify the best target cell line for our lentiviral system, we compared the infectivity of HIV-1–NL4.3–inGluc bearing SARS-CoV-2 S, along with that of SARS-CoV S and VSV-G, in a panel of cell lines, some of which were engineered to overexpress ACE2. Among these cells, we found that 293T/ACE2 cells consistently offered the most robust and consistent Gluc signals (Figure 1F), likely because of their highly proliferative nature compared with other cell lines. Accordingly, 293T/ACE2 cells were used in the experiments below.

### Processed SARS-CoV-2 S protein is incorporated into the lentiviral vector.

**Figure 2:**
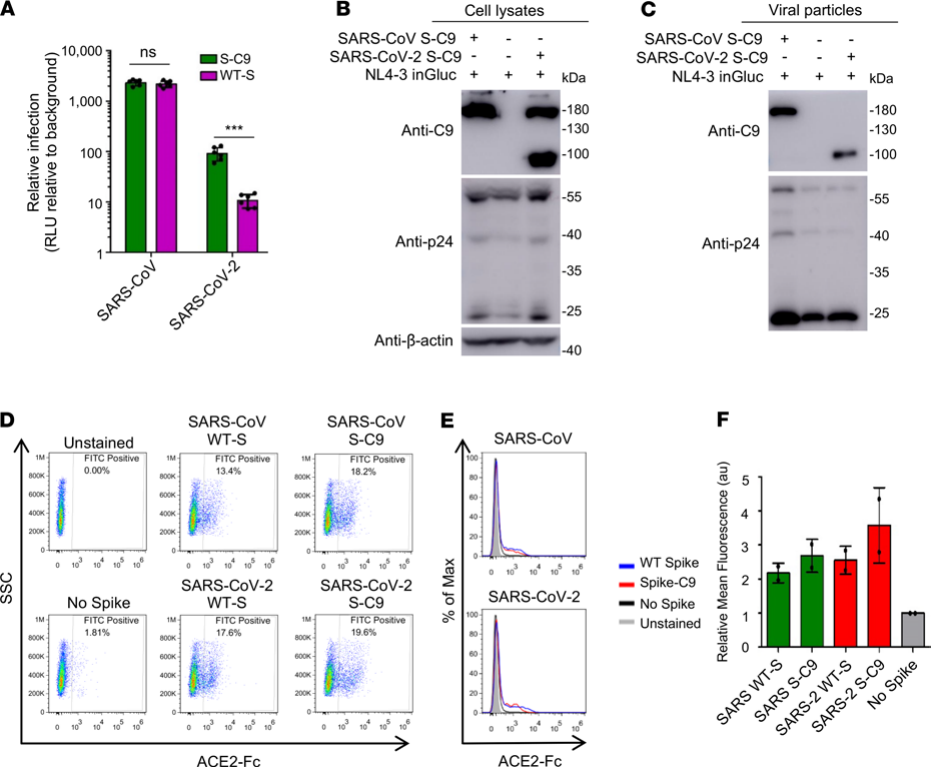
Comparison of HIV-1 inGluc pseudotypes bearing C9-tagged spikes of SARS-CoV or WTs. (**A**) Relative infectivity. Experiments were performed as described as Figure 1, B and C, except that either WT or C9-tagged spikes were used for virus production; *n* = 6. *P < 0.001, by 2-tailed *t* test. (**B** and **C**) Western blotting analysis of C9-tagged S protein expression in the virus-producing cells (**B**) and purified viral particles (**C**). Viral production was carried as described in Figure 1, and viral producer cells were lysed and analyzed by Western blotting using anti-C9, anti-p24, and/or anti–β-actin. (**D**–**F**) Virus-producing cells were digested with PBS-5 mM EDTA and incubated with 10 μg/mL sACE2-Fc for 2 hours; cells were washed 3 times, incubated with FITC-labeled anti-human Fc for 45 minutes, and analyzed by flow cytometry. (**D**) Representative cell populations analyzed for SARS-CoV and SARS-CoV-2; the percentage of positive cells for FITC anti-human Fc was shown. (**E**) Histogram analysis of virus-producing cells for SARS-CoV and SARS-CoV-2. (**F**) Relative mean fluorescence intensities of cells expressing indicated spikes; *n* = 2; no statistical analysis was performed. Note that cells transfected with an empty vector pCIneo served as negative control, the fluorescence intensity of which was set to 1. Data were analyzed as mean ± SD.

The S constructs used above for pseudotyping the HIV-NL4.3-inGluc vector contain C9 tags at their C-termini, which allowed us to determine and compare their expression in cells and in viral particles. Moreover, it is important to know the pseudotyping efficiency of C9-tagged spikes versus their corresponding WT counterparts. As shown in Figure 2A, the titer of the S-C9–bearing SARS-CoV-2 had a 5- to 10-fold-increased titer relative to the WT counterpart; however, S-C9–bearing SARS-CoV did not exhibit a difference from its WT, at least in the 293T/ACE2 cells. We then performed Western blotting to examine the expression of the S proteins in viral producer cells. We found that the C9-tagged SARS-CoV and SARS CoV-2 S proteins were expressed, with somewhat similar efficiency, although the S protein of SARS-CoV-2 was cleaved more efficiently compared with that of SARS-CoV (Figure 2B). Western blotting analysis of purified S-pseudotyped viral particles revealed that the S-C9 protein of SARS-CoV was more efficiently incorporated into HIV-1 lentiviral particles compared with that of SARS-CoV-2. Despite this, the latter was present in its processed form (Figure 2C).

Due to the lack of a common antibody allowing us to detect all forms of S proteins (WT and C9 for both SARS-CoV and SARS-CoV-2), we used a soluble form of ACE2–human Fc fusion protein (ACE2-hFc) and anti-hFc in flow cytometry to examine the expression of these S proteins on the surface of the viral producer cells. As shown in Figure 2, D–F, no significant differences were observed between these spikes, regardless of whether they were SARS-CoV, SARS-CoV-2, or C9-tagged variants. Altogether, these results demonstrate that the S glycoprotein of both SARS-CoV-2 and SARS-CoV can pseudotype the lentiviral vector, with SARS-CoV-2 exhibiting a lower pseudotyping efficiency than SARS-CoV. However, the presence of a C9 tag on the C-terminus of SARS-CoV-2 S substantially increases the titer of lentiviral pseudotypes bearing SARS-CoV-2 S.

### Application of inGluc-based lentiviral SARS-CoV-2 S pseudotypes for detecting SARS-CoV-2/COVID-19 neutralizing antibody and correlations with infectious virus–based plaque reduction.

**Figure 3:**
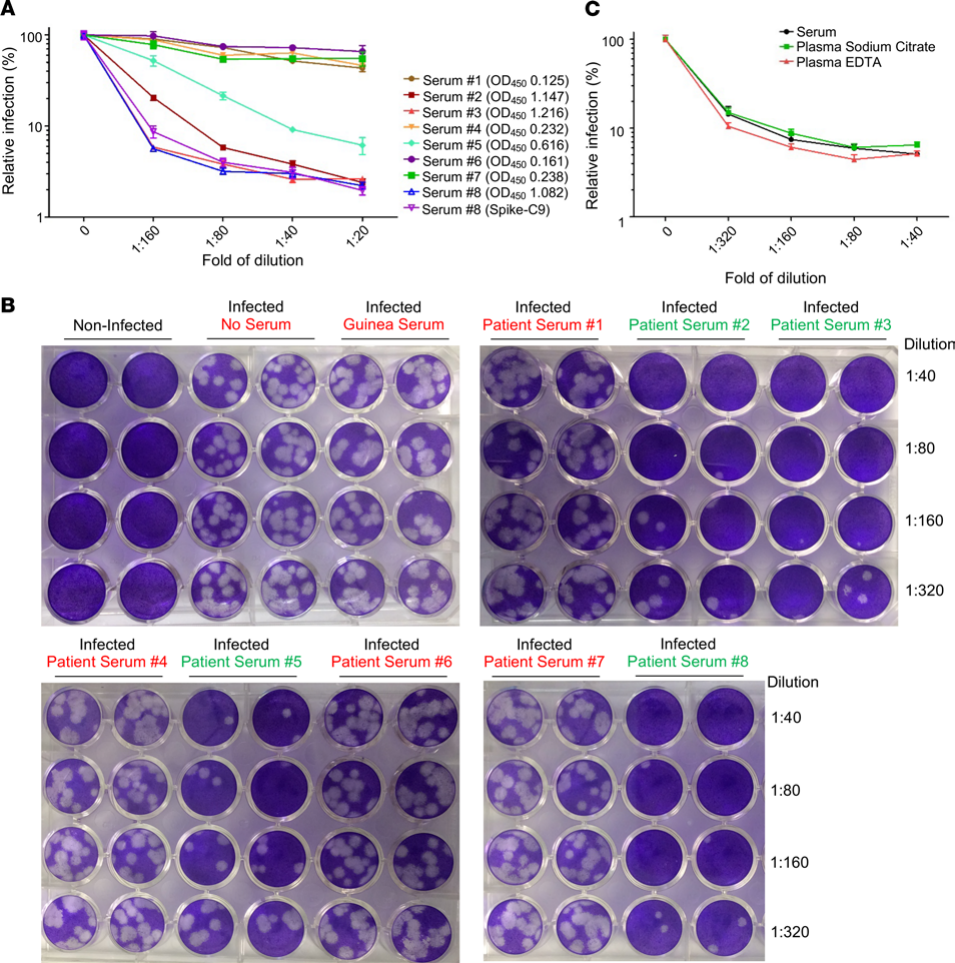
Neutralization of SARS-CoV-2 by COVID-19 patient sera. Validation of inGluc-based lentiviral pseudotypes using an authentic SARS-CoV-2 US-WA-1 strain. Note that all samples tested here and throughout the studies were blinded before testing. (**A**) A group of 8 blinded patient sera was tested for neutralization of the SARS-CoV-2 lentiviral S pseudotypes bearing C9-tagged SARS-CoV-2 S or WT. Note that only patient serum sample (sample 8) was tested for the WT spike, the pattern of which almost perfectly overlaps with that of C9-tagged spike; *n* = 3. The ELISA OD_450_ values of 8 samples are indicated for each number (cutoff, 0.40). (**B**) Results of infectious SARS-CoV-2 plaque-reduction neutralization assay for testing of 8 blinded samples. Vero-E6 cells were infected for 3 days with infectious SARS-CoV-2, pretreated with or without the indicated diluted sera. Cells were fixed and stained with 0.25% crystal violet for visualization of plaques; *n* = 3. Note that the BEI guinea pig antiserum to SARS-CoV did not inhibit SARS-CoV-2 infection. (**C**) Types of serum or plasma samples did not appear to affect the neutralization pattern generated by inGluc-based lentiviral pseudotypes bearing SARS-CoV-2 spike. Serum, sodium citrate-treated plasma, and EDTA-treated plasma from the same patient were used for SARS-CoV-2 S pseudotype neutralization. Data were analyzed as mean ± SD.

One goal of developing sensitive and convenient lentiviral pseudotypes bearing SARS-CoV-2 S is to evaluate the neutralizing antibody response in COVID-19–confirmed cases and SARS-CoV-2–exposed individuals. Toward this goal, we initially tested a small group of 8 blinded patient serum samples, with limited dilutions. Briefly, the samples were pretreated by heat inactivation at 56°C for 1 hour; serially diluted at 1:20, 1:40, 1:80, and 1:160; and then incubated with inGluc SARS-CoV-2 S pseudotypes at 37°C for 1 hour. The virus-sera mixtures were subsequently added to 293T/ACE2 cells to allow infection for 6 hours before being removed from target cells; culture media were collected from cells at 24 and 48 hours after infection and measured for Gluc luciferase activity. As shown in Figure 3A, samples 3 and 8 exhibited strong neutralizing activity, having > 90% inhibition of viral infectivity at 1:160 compared with samples 2 and 5, which showed 50% inhibition of viral infectivity between 1:80 and 1:160. In sharp contrast, negative control samples 4, 6, and 7, which were collected before September 2019 showed only background level of inhibition. Importantly, the neutralization pattern of patient sample 8 using WT S–bearing inGluc pseudotypes was identical to that using the S-C9–bearing pseudotypes (Figure 3A), indicating that the C9-tagged SARS-CoV-2 S faithfully mimics that of WT S in the neutralization assay. The virus neutralization results were consistent with N-based antibody ELISA OD_450_ values, with samples 2, 3, 5, and 8 having a range between 0.616 and 1.216 in contrast to samples 1, 2, 6, and 7, which were 0.125–0.238 (the ELISA cutoff was set to about 0.40) (Figure 3A). Results from this initial blinded testing gave us confidence that the inGluc-based SARS-CoV-2 S pseudotype virus neutralization assay is potentially useful for determining the neutralizing antibody titer in COVID-19 patients.

We further validated the inGluc-based virus neutralization assay with a PRVN assay using an infectious SARS-CoV-2 strain, USA-WA-1. As shown in Figure 3B, the numbers of SARS-CoV-2 plaques were greatly reduced by serum samples 2, 3, 5, and 8, with samples 3 and 8 being stronger than samples 2 and 5 in reducing plaques. In contrast, serum samples 1, 4, 6, and 7 had no impact on plaque numbers, which were similar to no serum treatment. These results correlated completely with that of the S pseudovirus–based virus neutralization assay shown in Figure 3A, although we were unable to calculate their exact titers due to limited sample volumes and dilutions. Of note, guinea pig serum against SARS-CoV (BEI Resources, catalog NR-10361) did not inhibit the plaque-forming activity of SARS-CoV-2, indicating that it has no cross-reactivity with SARS-CoV-2. Because all samples tested above were sera, we next evaluated if plasma, collected in different forms, could also be used in our S-bearing lentiviral neutralization assay. As shown in Figure 3C, serum, plasma-sodium citrate, and plasma-EDTA from 1 patient yielded an almost completely overlapping neutralization patterns, especially serum and plasma-sodium citrate. Thus, 3 forms of patient samples can be used for the inGluc-based SARS-CoV-2 neutralization assay.

### Cross-reactivity of antibodies between SARS-CoV-2 and SARS-CoV using inGluc-based lentiviral S pseudotypes.

**Figure 4:**
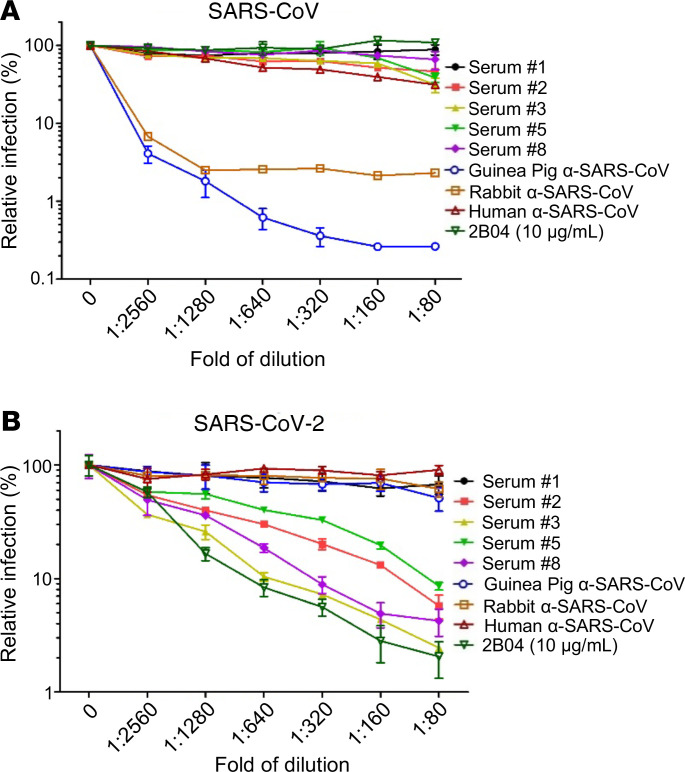
Examining the cross-reactivity of COVID-19 patient sera with SARS-CoV/SARS-CoV-2 S lentiviral pseudotypes. (**A** and **B**) The neutralization assay was carried out as described in Figures 3 and 4, except that HIV-1 inGluc pseudotypes bearing SARS-CoV S (**A**) were used in parallel with that of SARS-CoV-2 (**B**). In addition to 5 patient sera (samples 1, 2, 3, 5, and 8 from Figure 3A), stocks of polyclonal (guinea pig and rabbit) or monoclonal (human) antibodies against SARS-CoV obtained from BEI Resources were also tested; the exact concentrations of these antibodies are unknown. A mouse monoclonal antibody 2B04 against SARS-CoV-2, with a stock concentration of 10 μg/mL, was diluted to the same extent as the patient sera and other antibodies and tested; *n* = 3. Data were analyzed as mean ± SD.

Given the inconsistency of reports regarding cross-reactivity of SARS-CoV antibodies with SARS-CoV-2 (24–26), we extended our analyses using S-pseudotyped viruses. COVID-19 patient serum samples 2, 3, 5, and 8 and negative serum sample 1 shown in Figure 3A were further serially diluted from 1: 40 to 1:2560 and were tested for neutralizing antibody titers. As shown in Figure 4A, polyclonal rabbit (BEI, catalog NRC-777) and the guinea pig antisera against SARS-CoV showed potent inhibition of SARS-CoV infection, as would be expected; human monoclonal CR3022 (BEI, catalog 52392) also inhibited SARS-CoV infection, albeit with a much lower efficiency. None of the human COVID-19 patient serum samples, the negative control serum sample 1 collected before September 2019, or the mouse 2B04 monoclonal antibody against SARS-CoV-2 (34) showed any neutralizing activity against SARS-CoV. For SARS-CoV-2, we observed that the mouse SARS-CoV-2 monoclonal antibody 2B04 potently inhibited SARS-CoV-2 infection, with an IC_50_ of 3.007 ng/mL (Figure 4B). Consistent with data shown in Figure 3A, COVID-19 serum samples 2, 3, 5, and 8 showed varying degrees of SARS-CoV-2 neutralization, with samples 3 and 8 having an IC_50_ titer of 1:2639 and 1:2792, respectively (Figure 4B). Overall, these results demonstrate that the inGluc-based virus neutralization assay can reliably determine neutralizing antibody responses in COVID-19 patients and that sera or antibodies of human, mouse, rabbit, guinea pig, or nonhuman primates against SARS-CoV or SARS-CoV-2 do not strongly cross-react in our inGluc-based virus neutralization assays.

### High-throughput testing of neutralizing antibody in hospitalized COVID-19 patients, HCWs, and convalescent plasma donors.

**Figure 5:**
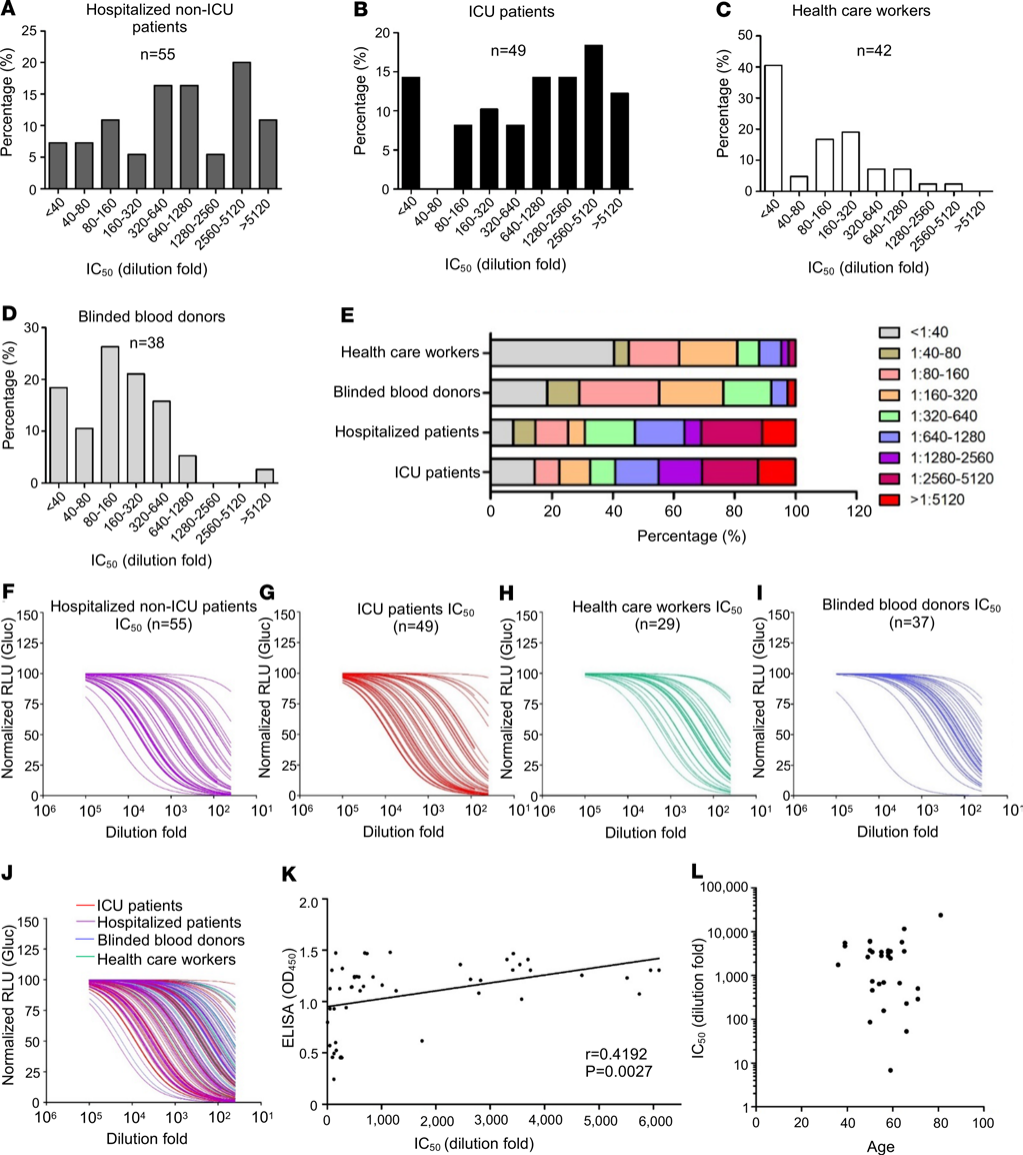
Evaluations of neutralizing antibody levels in COVID-19 hospitalized inpatients, ICU patients, health care workers, and convalescent plasma donors. Blinded serum samples were serially diluted and tested for neutralizing activity against lentiviral pseudotypes bearing SARS-CoV-2 S. (**A**–**E**) Ranges of neutralizing antibody titer IC_50_ in the 4 indicated groups (*x* axis); percent in each study group was plotted (*y* axis). (**F**–**J**) Neutralization curves of 4 different groups, as presented by the relative infectivity of SARS-CoV-2 S pseudotypes in the presence of indicated serum samples. The *y* axis indicates the relative viral infectivity by setting the viral infectivity without serum to 100%; the *x* axis indicates dilution fold of serum samples. (**K**) Correlational analysis of pseudovirus neutralization IC_50_ and N protein IgG antibody ELISA OD_450_ values; *r* = 0.4192, *P* = 0.0027 as indicated; *n* = 49. *r* = 0.4192, *P* = 0.0027, as indicated by correlation of XY analyses; *n* = 49. (**L**) Correlational analysis between pseudovirus neutralization IC_50_ and age; *n* = 30. Data were analyzed as mean ± SD.

By expanding this assay to a 96-well high-throughput format, we determined the neutralizing antibody response in a cohort study. The cohort was composed of 104 hospitalized patients — including 55 general hospitalized inpatients and 49 ICU patients — as well as 42 Ohio State Medical Center HCWs who were PCR-positive for SARS-CoV-2 and 38 blinded convalescent plasma donors. All of these samples were collected between April and May 2020. In parallel, 37 frozen plasma samples, either from other respiratory disease cases or other patients collected before September 2019, were tested. We found that, in general, COVID-19 patients, including hospitalized and ICU patients, had high titers of SARS-CoV-2 neutralizing antibodies, with ~20% (20 of 104) exhibiting a titer between 1:2560 and 1:5120, and with 12% (12 of 104) having a titer > 1:5120 (Figures 5, A, B, and E). Among these, ICU patients (~45.0%; 22 in 49) showed an increased percentage of higher neutralizing titers above 1:1280 as compared with general hospitalized inpatients (~36.0%, 20 of 55). We also noted that 14.5% (8 of 55) of general hospitalized inpatients and 14.3% of ICU patients (7 of 49) had no or low detectable neutralizing antibody (<1:80), which was close to the ~10% rate of previous reports (Figures 5, A, B, and E). In sharp contrast, although all HCWs were confirmed reverse transcription PCR–positive (RT-PCR–positive) for SARS-CoV-2 infection, ~40% of the HCWs (17 of 42) were negative for SARS-CoV neutralizing antibody, and 36% (15 of 42) showed an intermediate titer between 1:80 and 1:320, with only 5% (2 of 42) having a titer over 1:1280 (Figure 5, C and E). Notably, > 55% of blinded convalescent plasma donor samples (21 of 38) exhibited a titer lower than 1:160 (Figure 5, D and E), indicating that more than half of the blood donors did not qualify as convalescent donors for treatment of COVID-19 patients as per US Food and Drug Administration (FDA) guidelines (35). Nevertheless, ~26% of donor samples had a neutralization titer above 1:320, with 1 greater than 1:5120 (Figure 5, D and E).

Neutralization curves of all 4 groups of samples are presented in Figure 5, F–J, which again illustrates highly diverse levels of S pseudotype virus neutralizing antibody among these samples. By excluding those having an ELISA OD_450_ cutoff value of 0.40 or below (except 1, which is 0.24 from a PCR-positive HCW), we obtained a good correlation (*r* = 0.4192, *P* < 0.0027) between the S pseudotype virus neutralization titer and the ELISA OD_450_ values (Figure 5K), the latter of which was for the N protein of SARS-CoV-2 (36), indicating that the N-based ELISA can serve as a reliable method for large-scale screening of antibody responses to SARS-CoV-2 infection. Of note, there was no correlation between levels of neutralizing antibody with age (Figure 5L).

### Creation of a secreted Nluc–based lentiviral vector with improved stability and sensitivity for measuring SARS-CoV-2 neutralization.

**Figure 6:**
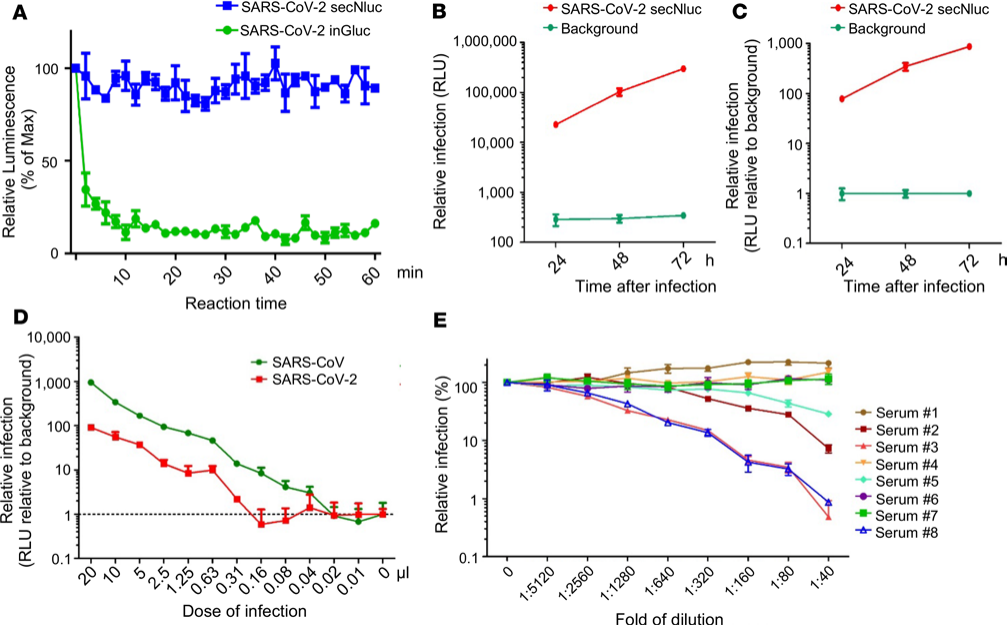
A secreted Nluc–based lentiviral SARS-CoV-2 S neutralization assay with improved stability and sensitivity, and its application in measuring SARS-CoV-2 neutralizing antibody levels in COVID-19 patients. 293T cells were transfected with lentiviral vector (pNL4.3 inGluc or pNL4.3 secNluc) along with the SARS-CoV-2 S-C9 plasmid. Media was 48 hours after transfection and used to infect 293T/ACE2 cells; luciferase activity was measured at indicated times to determine the viral infectivity; *n* = 3 for all experiments. (**A**) Stability of inGluc and secNluc luciferase signals measured over time. A total of 20 μL of *Gaussia* luciferase substrate or 20 μL of Nano Luciferase substrate were added simultaneously, and luminescence measurements were then read every 2 minutes for 60 minutes. Plotted are the luminescence reads relative to the 0 minutes time point, which was set to 100%; secNluc exhibited a signal that was more stable than the inGluc virus infected cells. (**B** and **C**) Infectivity of SARS-CoV-2 S secNluc pseudotypes. (**B**) The Nano-luciferase activity of culture medium harvested from virus-infected cells at indicated times. (**C**) Relative viral infectivity was plotted by setting the mock infection to 1.0. (**D**) Indicated amounts of viral supernatant were used to infect 293T/ACE2 cells seeded in 24-well plates, and 20 μL of supernatant from virus-infected cells was used to measure the secNluc activity as shown. The dashed line indicates the background luminescence. (**E**) Experiment was performed as described in the legends of Figure 3A and Figure 4B, except secNluc lentiviral pseudotypes were used for infection. Data were analyzed as mean ± SD.

While highly sensitive and convenient, one disadvantage of using Gluc as a reporter gene compared with firefly or other luciferase forms is the rapid decay of its signal during measurement. To alleviate this complication, we created an intron containing, secreted Nluc–based (secNluc-based) HIV-1–NL4.3 vector, in which the Gluc gene in the inGluc HIV-1 vector was replaced by a Nluc reporter containing an IL-6 secretion signal, and this secNluc reporter was also split by an intron. Nluc is about 100-fold brighter than firefly or renilla luciferase and produces high-intensity, glow-type luminescence; having an IL-6 secretive signal at its N-terminus renders this reporter secreted into culture medium, as is Gluc. We tested this vector with SARS-CoV-2 S pseudotyping, and we found that, as expected, the secNluc signal was much more stable, showing no apparent loss in signal over the 1-hour testing period. In contrast, the inGluc signal decayed rapidly, within a matter of minutes (Figure 6A). Importantly, this potentially new vector was pseudotyped by SARS-CoV-2 S as efficiently as that for the inGluc vector, yet it exhibited significantly more robust and increased luciferase signals. Even at 24 hours after infection, the secNluc signal of SARS-CoV-2 S pseudotypes were about 100-fold above the background level (Figure 6, B and C), which shortened the detection window from the prior 48 hours.

To further determine assay sensitivity, we carried out infection with endpoint-diluted secNluc peseudotypes, as we did for the inGluc vector (Figure 1D), and we observed that — in a typical experiment — as little as 0.31 μL of secNluc SARS-CoV-2 S pseudotypes showed an above-the-background level of Nluc activity at 24 hours after infection (Figure 6D), confirming that this potentially new vector indeed shortened the SARS-CoV-2 S pseudotype virus neutralization assay to within 24 hours. The estimated titers of secNluc SARS-CoV-2 and SARS-CoV S pseudotypes were about 1.6 × 10^5^ and about 1.3 × 10^6^ IUs per mL, respectively, a 2- to 4-fold increase compared with that from the inGluc-based system.

We next used the second-generation secNluc SARS-CoV-2 S pseudotype system to measure neutralizing antibody levels of COVID-19 patient sera, which had been determined by the inGluc S pseudoypes. We observed that samples 3 and 8 still exhibited the strongest neutralizing activity, with a calculated IC_50_ of 1:4189 and 1:2556, respectively. Samples 2 and 5 also showed strong neutralizing activities, with an IC_50_ between 1:1000 and 1:1800. This pattern of neutralizing activity was concordant with results obtained with the inGluc assay (Figure 3 and Figure 4). As expected, 4 negative control samples 1, 4, 6, and 7 showed no neutralizing activity (Figure 6E). Overall, these data demonstrate that the second-generation assay, leveraging the secNluc-based SARS-CoV-2 pseudotype virus, is rapid and has a significantly improved stability and sensitivity to accurately measure the neutralizing antibody levels of COVID-19 patients.

## Discussion

In this work, we described a sensitive and reliable SARS-CoV-2 S–bearing lentivirus inGluc neutralization assay that is validated by the authentic SARS-CoV-2 plaque-reduction assay. We evaluated the neutralizing antibody response in 4 groups of individuals with the potentially new pseudotype virus assay: general hospitalized COVID-19 inpatients, ICU patients, university HCWs exposed to SARS-CoV-2, and convalescent plasma donors. In general, we found that hospitalized COVID-19 patients — especially ICU patients, which were PCR confirmed — had a higher neutralizing antibody titer against the SARS-CoV-2 S pseudotype virus (50% > 1:640), some reaching > 1:5120, as compared with PCR-positive HCWs and convalescent plasma donors, the majority of whom had titers of < 1:640. However, the exact immunoglobulin subtypes that account for the observed neutralization for SARS-CoV-2, which will be addressed in future studies, is currently unclear. We also examined possible cross-reactivity of several reference monoclonal antibodies and polyclonal sera against SARS-CoV and SARS-CoV-2 using our assay, and we clearly ruled out cross-reactivity with the 2 closely related viruses. Importantly, none of the 8 COVID-19 patient sera showed any neutralization of SARS-CoV S pseudotype virus. Work is underway to generate lentiviral pseudotypes bearing various spikes from seasonal coronaviruses and to examine if SARS-CoV-2–positive sera may neutralize these viruses. Last, we were able to improve the inGluc-based SARS-CoV neutralization assay by modifying the lentiviral vector with a secNluc reporter, which makes the assay even more rapid and sensitive.

A principle of lentiviral pseudotyping, as with many other pseudotyping systems, is incorporation of viral envelope glycoproteins into the vector of interest (37). In this work, by using C9-tagged S constructs, we demonstrate that the S proteins of both SARS-CoV-2 and SARS-CoV are incorporated into the HIV-1–NL4.3–inGluc vector — yet with different efficiencies. The SARS-CoV-2 S protein appears to be less efficiently incorporated compared with that of SARS-CoV, which may account in part for the relatively lower infectivity of SARS-CoV-2 S pseudotypes. This occurs despite comparable surface expression of these S proteins on viral producer cells. Another interesting observation from this study is that the C9-tagged SARS-CoV-2 S shows ~10-fold higher titer over the WT S, yet there is no increase in titer between C9-tagged SARS-CoV S and its WT. Work is ongoing to further decipher the underlying mechanisms of these observations. Importantly, the increased titer of SARS-CoV-2 inGluc pseudotypes bearing C9-tagged S faithfully mimic that of WT in evaluating the neutralizing antibody response in COVID-19 patients and SARS-CoV-2–exposed individuals.

The inGluc-based and secNluc-based lentiviral SARS-CoV-2 S pseudotype virus neutralizing assays have several advantages over others that have been reported (18–22). First, the assays reported here use a naturally secreted Gluc, or a modified Nluc, which is secreted as a reporter, thus requiring no cell lysis or detachment of target cells before measurements of reporter gene expression (28). Moreover, culture media containing the secreted Gluc or Nluc can be harvested and measured at multiple times during the infection period, which greatly increases the flexibility, efficiency, and reproducibility of this assay. Second, our assay uses an intron in the sense genome of HIV-1 vector that splits the antisense Gluc or Nluc gene; only after viral infection of target cells, when the intron is spliced out, can the full-length Gluc or Nluc gene be generated, leading to their expression and detection (29, 30). This feature eliminates background luciferase activity possibly carried over from viral producer cells, which could otherwise confound data analyses. Third, the HIV-1–NL4.3–inGluc or –secNluc vector expresses gag-pol, in addition to accessory genes, allowing for a relatively high dose of S protein–coding plasmids to be cotransfected. This feature is particularly helpful, given the presence of a putative ER retention signal in the cytoplasmic tail of the SARS-CoV-2 S protein (38, 39), which intrinsically may restrict its expression on the plasma membrane of producer cells and its incorporation into the viral particles. Last, this assay is simple, rapid, and cost effective, because fewer procedures and reagents are needed compared with other reporter gene–based assays. These advantages make the inGluc- and secNluc-based virus neutralization assay particularly suitable for large-scale testing of COVID-19 serum or plasma from clinical cases, virus-exposed individuals, convalescent plasma donors and recipients, and vaccinated humans and animals, as well as clinical trial participants. In addition, the new assays can be used for high-throughput screens of monoclonal antibodies and inhibitors for SARS-CoV-2, as well as many emerging viral pathogens.

## Methods

### Constructs, reagents, and cell lines.

Constructs used for production of lentiviral pseudotypes included HIV-1–NL4.3–inGluc vector (29, 30), which was originally obtained from David Derse’s lab at NIH (National Cancer Institute, Frederick, Maryland, USA) and Marc Johnson’s lab at the University of Missouri (Columbia, Missouri, USA). Plasmids pcDNA3.1-SARS-CoV-S-C9 and pcDNA3.1-SARS-CoV2-S-C9 (31), which encodes codon-optimized full-length spikes tagged with C9 at the C-terminus, were from Fang Li’s lab at the University of Minnesota (St. Paul, Minnesota, USA). Plasmids pcDNA-SARS-CoV-S and paH-SARS-CoV-2-S (40), which encodes codon-optimized full-length spikes, were from Jason McLellan’s lab at the University of Texas-Austin (Austin, Texas, USA). The mouse monoclonal antibody 2B04 against SARS-CoV-2 was a gift from Ali Ellebedy at Washington University (St. Louis, Missouri, USA) (34). Antibodies used for Western blotting included anti-C9 (anti-rhodopsin) (Santa Cruz Biotechnology Inc., 57432), anti-p24 (Abcam, ab63917), anti–β-actin (MilliporeSigma, A1978), and secondary antibodies anti–mouse IgG (MilliporeSigma, A5278) and anti–rabbit IgG (MilliporeSigma, A9169). Secondary antibodies used for flow cytometry included FITC-conjugated anti–human IgG-Fc (MilliporeSigma, F9512).

HEK293T (ATCC CRL-11268, research resource identifier [RRID]: CVCL_1926), HeLa (ATCC, CCL-2, RRID: CVCL_0030), HTX (a subclone of HT1080), A549 (ATCC, CCL-185, RRID: CVCL_0023), and Huh7.5 (RRID: CVCL_7927) cells were grown in DMED (MilliporeSigma, D5796), supplemented with 1% penicillin/streptomycin (MilliporeSigma, P4333) and 10% (vol/vol) FBS (Thermo Fisher Scientific, 26140-079). Calu-3 (ATCC, gift of gift of Estelle Cormet-Boyaka at The Ohio State University) were grown in Eagle’s Minimum Essential medium (EMEM) (ATCC, 30-2003), supplemented with 1% penicillin/streptomycin (MilliporeSigma, P4333) and 10% (vol/vol) FBS (Thermo Fisher Scientific, 26140-079). The HEK293T/ACE2 cell line is a gift from Fang Li at the University of Minnesota. HeLa, A549, HTX, and Huh7.5 cells stably expressing ACE2 were generated by transduction of pLenti-GFP vectors expressing ACE2 (OriGene, RC208442L4), followed by puromycin selection (1 μg/mL) for 6 days. All cell lines used were maintained at 37°C, 5% CO_2_. Authentic SARS-CoV-2 virus US-WA-1 strain was obtained from BEI Resources (catalog NR-52281).

### Creation of a secreted intron-bearing Nano-Luc lentiviral vector.

The secNluc construct (Promega, N1031, gift from Walther Mothes, Yale University, New Haven, Connecticut, USA) contains the Nluc construct with an N-terminal IL-6 secretion signal, allowing the Nluc to be secreted, similarly to Gluc. N-terminal and C-terminal portions of this construct were cloned to include the same β-globin intron as the Gluc construct, also in the opposite orientation. This fragment was then introduced into the original pNL4.3 inGluc vector, with the inGluc cassette removed. This produced a similar construct containing a sense orientation HIV-1 gag-pol gene and an antisense orientation secNluc gene containing a sense orientation β-globin intron. This allows for the production of pseudotyped virus containing the secNluc gene without the production of secNluc in the virus-producing cells, as was the case for our pNL4.3 inGluc construct.

### Patient samples and specimens.

All samples were deidentified specimens from a clinical laboratory, and handling of these samples was under an approved IRB protocol (OSU 2020H0228). Plasma and serum were collected from hospitalized COVID-19 inpatients or ICU patients, OSU HCWs, and blinded convalescent plasma donors and analyzed in a blinded manner. Plasma was prepared using EDTA, lithium heparin, or sodium citrate anticoagulated blood samples while serum was prepared using Gold top serum-separator tubes (Becton Dickinson) or Red top clotting tubes (Becton Dickinson). Samples were incubated for 24 hours at room temperature to allow plasma/serum to separate. Then, plasma/serum was isolated and frozen (–20°C). Samples found to be severely hemolytic were rejected.

### Production of inGluc- or secNluc-based lentiviral pseudotypes bearing the S protein of SARS coronaviruses and viral infection.

For inGluc– or secNluc-based pseudotyped lentiviral production, we transfected HEK293T cells with HIV-1–NL4.3–inGluc or –secNluc vector plus a plasmid expressing the S protein or VSV-G in a 2:1 ratio using polyethylenimine (PEI). Supernatants were harvested at 24, 48, and 72 hours after transfection; aliquoted; and stored at –80°C. For viral infection, we added appropriate amounts of virus onto target cells and incubated plates (37°C) for 6 hours before changing media; we then measured Gluc at 24, 48, and 72 hours after infection. One IU was defined as the luciferase readout equivalent above the background threshold in cells infected with lentiviral pseudotypes bearing no S protein.

### SARS-CoV and SARS-CoV-2 S pseudotype virus neutralization assay.

In the virus neutralization assay, we used 100 μL virus for each well in 96-well plates. Virus was incubated with patient or control serum or plasma, monoclonal antibodies, or polyclonal antisera for 1 hour at 37°C. These included guinea pig antisera (BEI Resources, NR-10361), rabbit antisera (BEI Resources, NRC-777), human monoclonal CR3022 (BEI Resources, NR-52392), and mouse monoclonal 2B04 (a gift from Ali Ellebedy, Washington University). Media was then removed from seeded HEK293T/ACE2 cells and replaced with the virus/serum or plasma mixture. The infection was allowed to proceed for 6 hours at 37°C before changing to fresh media. Gluc or Nluc activity was measured at 24, 48, and 72 hours after media change. For luciferase measurement, unless specified, 20 μL of supernatant were collected from each well and transferred to a white nonsterile 96-well plate. To each well, 20 μL of Gluc substrate (0.1M Tris [MilliporeSigma, T6066] pH 7.4, 0.3M sodium ascorbate [Spectrum, S1349], 10 μM coelenterazine [GoldBio, CZ2.5]) or Nluc substrate (Promega, N1110) was added, and luminescence was immediately read by a plate reader.

### Infectious virus plaque reduction neutralization assay.

Serum samples were diluted in DMEM, mixed with 80 TCID50 SARS-CoV-2, and incubated for 1 hour at 37°C. Serum/virus mixtures were then used to infect confluent Vero-E6 cells for 1 hour at 37°C. The serum/virus was then removed from cells and replaced with 0.3% agarose (MilliporeSigma, A9539) in DMEM/4% FBS and incubated for 72 hours at 37°C. Cells were then fixed with 4% paraformaldehyde in PBS and stained with 0.25% crystal violet (MilliporeSigma, C0775) in 20% ethanol/water for visualization of plaques.

### Flow cytometry analysis of S expression on the cell surface by soluble ACE2-hFc.

HEK293T cells were transfected with 1000 ng S-C9 or WT S constructs with PEI. After 36 hours after transfection, cells were washed with PBS (MilliporeSigma, D5652-1L), detached with PBS containing 5 mM EDTA (Bio-Rad, 161-0729) for 10 minutes, washed twice with cold PBS plus 2% FBS, and incubated with soluble ACE2-hFc proteins (10 μg/mL, a gift from Jason McLellan’s lab at the University of Texas at Austin) for 2 hours. After 3 washes with PBS plus 2% FBS, cells were incubated with FITC-conjugated anti–human IgG (1:200, MilliporeSigma, F0257) secondary antibodies for 1 hour. Cells were washed 3 times with cold PBS plus 2% FBS and fixed with 3.7% formaldehyde for 10 minutes and analyzed by flow cytometry.

### Western blotting.

Western blotting was performed as previously described (41, 42). In brief, cells were collected and lysed in RIPA buffer (50 mM Tris [MilliporeSigma, T6066] pH 7.5, 150 mM NaCl [Fisher Chemical, S271-500], 1 mM EDTA [Bio-Rad, 161-0729], Nonidet P-40 [Thermo Fisher Scientific, 85124], 0.1% SDS [MilliporeSigma, L3771-500G], and protease inhibitor cocktail [MilliporeSigma, P8340]), which disrupts membrane-associated proteins. Cell lysates were clarified by centrifugation for 10 minutes, 12,000*g* at 4°C, and boiled at 100°C for 10 minutes with SDS loading buffer containing 2-Mercaptoethanol. Treated samples were resolved on 10% SDS-PAGE gels, transferred to PVDF membranes (Bio-Rad, 162-0177), and probed with primary antibodies.

### ELISA.

ELISA was performed by using the EDI Novel Coronavirus COVID-19 N protein IgG ELISA Kit (EDI, KT-1032) following manufacturer’s protocol. Briefly, 100 μL of 1:100 diluted serum/plasmid were added to microplates coated in SARS-CoV-2 antigen (N), and plates were incubated for 30 minutes at room temperature. Following wash steps, wells were treated with 100 μL of HRP labeled anti–human-IgG tracer antibody (EDI, 31220), incubated at room temperature for 30 minutes, and again washed. Then, 100 μL of ELISA HRP substrate (EDI, 10020) was added and incubated for 20 minutes at room temperature. Finally, 100 μL stop solution (EDI, 10030) was added, and absorbance at 450 nm was read with a spectrophotometric plate reader using Gen 5 software.

### Statistics.

Data were analyzed as mean ± SD. Statistical analyses were performed using 2-tailed *t* tests in GraphPad Prism 5.0; A *P* value of less than 0.05 was considered significant. For calculating IC_50_, nonlinear regression of XY analyses were performed and fitted with inhibition curve. For ELISA OD_450_ and IC_50_ correlation analyses, correlation of XY analyses were performed.

### Study approval.

The study was approved by OSU’s IRB (no. 2020H0228).

## Author contributions

SLL conceived and led the project. CZ and JPE the performed majority of the presented experiments. RP and SP collected patient’s samples and/or performed ELISA. PQ and YMZ produced virus and performed infections. RR, LHS, and JY performed infectious virus plaque reduction assay. RTR, JY, RKM, LS, EO, and GL contributed to data analyses and discussion. CZ, JPE, and SLL wrote the paper, which was edited by LS, EO, RKM, and JY.

## Version 1 10/09/2020

In-Press Preview

## Version 2 11/19/2020

Electronic publication

## References

[1] [No authors listed]. Coronavirus Disease (COVID-19): Situation Report. World Health Organization. https://www.who.int/emergencies/diseases/novel-coronavirus-2019/situation-reports Accessed October 15, 2020

[2] Wu F (2020). A new coronavirus associated with human respiratory disease in China. Nature.

[3] Hachim A, et al. Beyond the Spike: identification of viral targets of the antibody response to SARS-CoV-2 in COVID-19 patients. medRxiv. 10.1101/2020.04.30.2008567 Published May 22, 2020. Accessed October 15, 2020

[4] Luchsinger LL, et al. Serological Assays Estimate Highly Variable SARS-CoV-2 Neutralizing Antibody Activity in Recovered COVID19 Patients [published ahead of print September 11, 2020]. *J Clin Microbiol*. https://doi.org 10.1128/JCM.02005-20 10.1128/JCM.02005-20 PMC7685895 32917729

[5] Robbiani DF, et al. Convergent antibody responses to SARS-CoV-2 infection in convalescent individuals. bioRxiv. 10.1101/2020.05.13.092619 Published May 22, 2020. Accessed October 15, 2020 PMC7442695 32555388

[6] Wajnberg A, et al. Humoral response and PCR positivity in patients with COVID-19 in the New York City region, USA: an observational study [published ahead of print September 25, 2020]. Lancet Microbe . 10.1016/S2666-5247(20)30120-8 PMC7518831 33015652

[7] Cutts FT, Hanson M (2016). Seroepidemiology: an underused tool for designing and monitoring vaccination programmes in low- and middle-income countries. Trop Med Int Health.

[8] Jiang S, Hillyer C, Du L (2020). Neutralizing Antibodies against SARS-CoV-2 and Other Human Coronaviruses. Trends Immunol.

[9] Pickering S (2020). Comparative assessment of multiple COVID-19 serological technologies supports continued evaluation of point-of-care lateral flow assays in hospital and community healthcare settings. PLoS Pathog.

[10] Wu LP (2007). Duration of antibody responses after severe acute respiratory syndrome. Emerg Infect Dis.

[11] Callow KA, Parry HF, Sergeant M, Tyrrell DA (1990). The time course of the immune response to experimental coronavirus infection of man. Epidemiol Infect.

[12] Duan K (2020). Effectiveness of convalescent plasma therapy in severe COVID-19 patients. Proc Natl Acad Sci U S A.

[13] Cheng Y (2005). Use of convalescent plasma therapy in SARS patients in Hong Kong. Eur J Clin Microbiol Infect Dis.

[14] Chen L, Xiong J, Bao L, Shi Y (2020). Convalescent plasma as a potential therapy for COVID-19. Lancet Infect Dis.

[15] Bloch EM (2020). Deployment of convalescent plasma for the prevention and treatment of COVID-19. J Clin Invest.

[16] Chen X (2020). Human monoclonal antibodies block the binding of SARS-CoV-2 spike protein to angiotensin converting enzyme 2 receptor. Cell Mol Immunol.

[17] Seydoux E (2020). Analysis of a SARS-CoV-2-Infected Individual Reveals Development of Potent Neutralizing Antibodies with Limited Somatic Mutation. Immunity.

[18] Schmidt F (2020). Measuring SARS-CoV-2 neutralizing antibody activity using pseudotyped and chimeric viruses. J Exp Med.

[19] Crawford KHD (2020). Protocol and Reagents for Pseudotyping Lentiviral Particles with SARS-CoV-2 Spike Protein for Neutralization Assays. Viruses.

[20] Nie J (2020). Establishment and validation of a pseudovirus neutralization assay for SARS-CoV-2. Emerg Microbes Infect.

[21] Hu J, Gao Q, He C, Huang A, Tang N, Wang K. Development of cell-based pseudovirus entry assay to identify potential viral entry inhibitors and neutralizing antibodies against SARS-CoV-2 [published ahead of print July 13, 2020]. Genes Dis . 10.1016/j.gendis.2020.07.006 PMC7366953 32837985

[22] Muruato AE (2020). A high-throughput neutralizing antibody assay for COVID-19 diagnosis and vaccine evaluation. Nat Commun.

[23] Wu F (2020). Evaluating the Association of Clinical Characteristics With Neutralizing Antibody Levels in Patients Who Have Recovered From Mild COVID-19 in Shanghai, China. JAMA Intern Med.

[24] Lv H (2020). Cross-reactive Antibody Response between SARS-CoV-2 and SARS-CoV Infections. Cell Rep.

[25] Wang Y (2020). Kinetics of viral load and antibody response in relation to COVID-19 severity. J Clin Invest.

[26] Ou X (2020). Characterization of spike glycoprotein of SARS-CoV-2 on virus entry and its immune cross-reactivity with SARS-CoV. Nat Commun.

[27] Pinto D (2020). Cross-neutralization of SARS-CoV-2 by a human monoclonal SARS-CoV antibody. Nature.

[28] Goerke AR, Loening AM, Gambhir SS, Swartz JR (2008). Cell-free metabolic engineering promotes high-level production of bioactive Gaussia princeps luciferase. Metab Eng.

[29] Mazurov D, Ilinskaya A, Heidecker G, Lloyd P, Derse D (2010). Quantitative comparison of HTLV-1 and HIV-1 cell-to-cell infection with new replication dependent vectors. PLoS Pathog.

[30] Yu J (2015). IFITM Proteins Restrict HIV-1 Infection by Antagonizing the Envelope Glycoprotein. Cell Rep.

[31] Shang J (2020). Structural basis of receptor recognition by SARS-CoV-2. Nature.

[32] Shi R (2020). A human neutralizing antibody targets the receptor-binding site of SARS-CoV-2. Nature.

[33] Li W (2003). Angiotensin-converting enzyme 2 is a functional receptor for the SARS coronavirus. Nature.

[34] Alsoussi WB (2020). A Potently Neutralizing Antibody Protects Mice against SARS-CoV-2 Infection. J Immunol.

[35] [No authors listed]. Food Drug Administration. Recommendations for Investigation COVID-19 Convalescent Plasma. https://www.fda.gov/vaccines-blood-biologics/investigational-new-drug-ind-or-device-exemption-ide-process-cber/recommendations-investigational-covid-19-convalescent-plasma Updated September 2, 2020. Accessed October 15, 2020

[36] Krüttgen A, Cornelissen CG, Dreher M, Hornef M, Imöhl M, Kleines M (2020). Comparison of four new commercial serologic assays for determination of SARS-CoV-2 IgG. J Clin Virol.

[37] Cronin J, Zhang XY, Reiser J (2005). Altering the tropism of lentiviral vectors through pseudotyping. Curr Gene Ther.

[38] McBride CE, Li J, Machamer CE (2007). The cytoplasmic tail of the severe acute respiratory syndrome coronavirus spike protein contains a novel endoplasmic reticulum retrieval signal that binds COPI and promotes interaction with membrane protein. J Virol.

[39] Lontok E, Corse E, Machamer CE (2004). Intracellular targeting signals contribute to localization of coronavirus spike proteins near the virus assembly site. J Virol.

[40] Wrapp D (2020). Cryo-EM structure of the 2019-nCoV spike in the prefusion conformation. Science.

[41] Côté M, Zheng YM, Li K, Xiang SH, Albritton LM, Liu SL (2012). Critical role of leucine-valine change in distinct low pH requirements for membrane fusion between two related retrovirus envelopes. J Biol Chem.

[42] Liu SL, Lerman MI, Miller AD (2003). Putative phosphatidylinositol 3-kinase (PI3K) binding motifs in ovine betaretrovirus Env proteins are not essential for rodent fibroblast transformation and PI3K/Akt activation. J Virol.

